# Editorial: Innovative applications of sequencing technologies in plant science

**DOI:** 10.3389/fpls.2022.1058347

**Published:** 2022-10-26

**Authors:** Ruslan Kalendar, Charles Hunter, Vladimir Orbovic

**Affiliations:** ^1^ Helsinki Institute of Life Science HiLIFE, Biocenter 3, University of Helsinki, Helsinki, Finland; ^2^ National Laboratory Astana, Nazarbayev University, Astana, Kazakhstan; ^3^ Chemistry Research Unit, United States Department of Agriculture (USDA) Agricultural Research Service, Gainesville, FL, United States; ^4^ Citrus Research and Education Center, University of Florida/Institute of Food and Agricultural Sciences (IFAS), Lake Alfred, FL, United States

**Keywords:** DNA metabarcoding, PCR technologies, plant genetics, genome walking, high-throughput sequencing

Sequencing technologies have led the way in a life sciences revolution that has unlocked previously impossible opportunities to examine the mysteries of life at the fundamental level of DNA (Amarasinghe et al., 2020). High-throughput sequencing technologies have enabled incredible gains in accuracy and efficiency for analysis of DNA and made possible applications on a much larger scale than was previously achievable (Costessi et al., 2018). Applications of high-throughput sequencing that have been particularly helpful in plant sciences include population screening and targeting identified traits of interest. Despite major gains in sequencing technology, its full potential to explore genetic information has not yet been realized. Emerging research continues to develop innovative ways to use high-throughput sequencing technologies to better understand the genetic nature of plants.

Plant mutagenesis is used to generate new gene variants (Sikora et al., 2011) and is useful both in plant breeding and studies of gene function. The combination of the introduction of technologies based on high-throughput sequencing and advanced genetic screening has significantly improved the discovery of genes in large-genome organisms, which includes many cultivated plants, such as barley. The precise roles of most genes in cultivated plants remains unknown, so mutant collection provide a valuable resource for studying the genetic basis of a broad spectrum of sophisticated biological systems. Li et al., demonstrate that a combination of low-resolution genetic mapping with genome-wide resequencing coupled with functional benchmarking analysis can identify potential candidate genes located even in recombination-poor regions of the complex barley genome. As an example, a gene (HvClpC1) was identified as a candidate for the barley yellow-green variegation mutant luteostrians mutant using these approaches.

Plant species identification and authentication approaches based on DNA metabarcoding using next-generation sequencing can be successfully used to confirm species identification of herbs and other commercial products. Raclariu-Manolică et al., used DNA metabarcoding on Ion Chef System in combination with traditional chemical methods analyze DNA from 62 products, containing basil, oregano, and paprika collected from different retailers and importers in Norway as an example of quality control capability of DNA sequencing approaches. This integration of next-generation sequencing-based DNA metabarcoding with a set of analytical tools for monitoring the quality of fresh and/or processed plant foods improves product quality and consumer confidence.

Multiple strategies for targeting the capture of unknown genomic sequences contiguous with known DNA regions are based on multi-step variants of PCR methods. These genome walking (GW) strategies (Leoni et al., 2011) are fast and straightforward and eliminate the need for construction of multi-step and technically challenging genomic libraries. Designing at least one sequence-specific primer (SSP) that anneals to the target sequence of interest and pairing with a walking random primer is a general principle of all these methods. However, a limitation of all genome walking methods has been the development of a universal and efficient walking random primer and the selection of optimal PCR cycling conditions. The use of a degenerate walking random primer for complex genomic DNA can lead to nonspecific amplification. One possible solution to this limitation is to use thermal asymmetric interlaced PCR (TAIL-PCR) method (Jia et al., 2017), wherein three sequential PCR rounds using nested SSPs and a shorter random degenerate primer can lead to greater specificity. Peng et al., attempted to locate the insertion position of the exogenous sequence (G10evo-5-enolpyrul-shikimate-3-phosphate synthase and Cry1Ab/Cry2Aj) in for SK12-5 transgenic maize line by using the TAIL-PCR and next-generation Illumina sequencing technology. In order to locate the fine-scale insertion position in SK12-5, these authors combined the methods of genetic mapping and nanopore-based sequencing technology. Using nanopore sequencing and a specialized software allowed the precise localization of T-DNA insertion within the genome of the transgenic SK12-5 line. This study demonstrates that the combined genetic mapping method and Oxford Nanopore sequencing technology can be used to identify insertion positions of transgenic sequences in genetically modified plants with large genomes.

Recently, a rapid palindromic sequence-targeted PCR (PST-PCR) assay has been developed that balances sensitivity and specificity (Kalendar et al., 2019). This PST-PCR technique is a novel walking primer design that enables annealing in both directions on a short palindromic sequence, for example, to type II restriction endonuclease palindromic recognition site (e.x., PstI, HindIII, etc). In the new version of this PST-PCR technology (called PST-PCR v.2) developed by Kalendar et al., following the first round of PCR, which uses a combination of one sequence-specific primer with one walking primer, a second round of PCR uses only a single universal tail primer that attaches both to the sequence-specific primer and to the walking primer. This is a major benefit of PST-PCR v.2 since utilizing one universal tail primer in GW processes involving various templates is highly suitable for simultaneous work with multiple samples. This approach can be applied beyond the classical task of GW for genotyping studies in population genetics and as an alternative to amplified fragment length polymorphism (AFLP) (Vos et al., 1995) or targeted next-generation sequencing. In this study, the utility of PST-PCR v.2 is used to analyze the variability associated with Ac transposon integration sites in the maize (*Zea mays*) genome (Sharma et al., 2021).

In summary, the research collected on this Research Topic highlights some important new applications of high-throughput sequencing technologies in Plant Science – such as in genetic mapping, the identification and characterization of candidate genes, innovative use of DNA metabarcoding, expansion of PCR technologies, and novel combinations of sequence-based technologies. All of these approaches can be leveraged to solve problems and answer questions in plant sciences, and thus help improve the planet’s health.

## Author contributions

RK and CH prepared the draft. All authors listed have made a substantial, direct, and intellectual contribution to the work and have approved it for publication.

## Funding

This work was supported by the Science Committee of the Ministry of Education and Science of the Republic of Kazakhstan (AP14869076) to RK and by the United States Department of Agriculture (USDA)-Agricultural Research Service Project number 6036-11210-001-00D to CH.

## Acknowledgments

We thank all authors and reviewers for their contributions to this Research Topic and for the support of the editorial office.

## Conflict of interest

The authors declare that the research was conducted in the absence of any commercial or financial relationships that could be construed as a potential conflict of interest.

The use of trade name, commercial product or corporation in this publication is for the information and convenience of the reader and does not imply an official recommendation, endorsement or approval by the USDA or the Agricultural Research Service for any product or service to the exclusion of others that may be suitable. USDA is an equal opportunity provider and employer.

## Publisher’s note

All claims expressed in this article are solely those of the authors and do not necessarily represent those of their affiliated organizations, or those of the publisher, the editors and the reviewers. Any product that may be evaluated in this article, or claim that may be made by its manufacturer, is not guaranteed or endorsed by the publisher.
